# Nanoscale measurement of the power spectral density of surface roughness: how to solve a difficult experimental challenge

**DOI:** 10.1186/1556-276X-7-174

**Published:** 2012-03-07

**Authors:** Juan Francisco González Martínez, Inés Nieto-Carvajal, José Abad, Jaime Colchero

**Affiliations:** 1 Instituto Universitario de Investigación en Óptica y Nanofísica, Campus de Espinardo, Universidad de Murcia, E-30100 Murcia, Spain

## Abstract

In this study, we show that the correct determination of surface morphology using scanning force microscopy (SFM) imaging and power spectral density (PSD) analysis of the surface roughness is an extremely demanding task that is easily affected by experimental parameters such as scan speed and feedback parameters. We present examples were the measured topography data is significantly influenced by the feedback response of the SFM system and the PSD curves calculated from this experimental data do not correspond to that of the true topography. Instead, either features are "lost" due to low pass filtering or features are "created" due to oscillation of the feedback loop. In order to overcome these serious problems we show that the interaction signal (error signal) can be used not only to quantitatively control but also to significantly improve the quality of the topography raw data used for the PSD analysis. In particular, the calibrated error signal image can be used in combination with the topography image in order to obtain a correct representation of surface morphology ("true" topographic image). From this "true" topographic image a faithful determination of the PSD of surface morphology is possible. The corresponding PSD curve is not affected by the fine-tuning of feedback parameters, and allows for much faster image acquisition speeds without loss of information in the PSD curve.

## 1 Introduction

The nanoscale surface morphology determines a wealth of phenomena which are important for fundamental science as well as for technological applications [1-3]. As is well known, surface roughness is a basic parameter in tribology [4,5], adhesion phenomena [2,6], the internal 3-D morphology of nanostructural functional materials [7], wetting properties of surfaces [8-11], optical reflectivity [12,13] as well as a wealth of biological processes [8,14]. A precise and reproducible measurement of surface roughness is therefore a key issue for basic science as well as for engineering applications [15]. Accordingly, important efforts have been undertaken in this field ranging from the development of suitable instruments to the normalization and traceability of length measurements. Traditionally, surface roughness has been measured using profilometers [15,16], although optical instruments have proven to be very powerful tools as well [17,18]. With the invention of the scanning tunneling microscope (STM) [19] and later, the scanning force microscope (SFM) [20], it has been possible to determine the morphology of surfaces down to the nanometer and even the atomic scale. Accordingly STM and SFM have been widely used for nanoscale roughness characterization [21-23].

The morphology of surfaces can be described using a variety of parameters, the root mean square (RMS) roughness is surely the most common one [24,25]. In addition, other parameters such as Skewness and Kurtosis can also be used to characterize a surface. Unfortunately, these parameters do not describe the morphology of surfaces in a sufficiently accurate way. This can be understood already on very simple arguments: if we assume that a surface has been discretized using *n *× *n *image points, the whole information content of the surface is reduced to one single value if only the RMS value of the surface is measured. Surfaces with very different morphology--and thus very different behavior with regard to tribology or adhesion--may have the same RMS value of surface roughness. Even worse, it can be shown that for many interesting surfaces the RMS roughness depends on the length scale used for the measurement; that is, as the size of an image is increased, the measured RMS also increases. The RMS-value of surface roughness is therefore not a scale invariant quantity. The precise description of surface morphology therefore calls for more sophisticated tools. As discussed in more detail elsewhere, the power spectral density (PSD) of surface roughness is such a tool [2,26]. PSD in combination with SFM is an invaluable tool in nanoscale science that should be further developed to really exploit all its possibilities [7,22,27-29].

Essentially the PSD describes the mean surface roughness at each length scale in a given image. Typically an image with *n *× *n *points results in a PSD curve with *n*/2 points. Evidently, even though the reduction of information content is quite high, the reduction is much less as compared to the case where only the RMS is computed. Interestingly, for many surfaces the roughness varies in a well defined way as the length scale is varied (so-called self afine surfaces). In this case the PSD curve is particularly simple since the relation log [PSD(*λ*)] versus log(*λ*), where *λ *is the wavelength of the surface roughness, is linear: log [PSD(*λ*)] versus log(*λ*) = *s *· log(*λ*) + *b *(for a detailed discussion about this matter, see [2]).

From a theoretical point of view, description of surfaces using PSD curves is a very powerful tool and is the basics for modern approaches relating tribology and adhesion phenomena to microscopic and nanoscale properties of surfaces [2]. Unfortunately, experimental determination of nanoscale PSD is quite demanding. Due to the compressing properties of the logarithm a large number of image points are needed to have a significant amount of data points for the horizontal axis (log(*λ*)). Moreover, since the relevant magnitude of the the vertical axis is also logarithmic (log [PSD(*λ*)]) a large dynamic range for the height measurement is also required, that is, very large as well as very small height differences have to be acquired equally well. Data adquisition in scanning probe microscopy (SPM) is sequential and thus inherently slow as compared to other imaging techniques which are generally based on parallel processing (as in most optical microscopes). As is well known, Scanning Probe techniques are based upon a very short range of interaction between the sample to be analyzed and a sharp tip used as probe. In order to obtain surface morphology, the tip is scanned over the sample while a feedback loop is used to keep tip-sample interaction at a constant value. As the tip moves over the sample, this feedback adjusts the (absolute) height of the tip in order to compensate for height variations of the sample surface. Correct adjustment of the feedback parameters is fundamental for the acquisition of good topography raw data: slow feedback will result in "smoothing" of topography data, which would effectively imply low pass filtering and a loss of high frequency roughness, while fast feedback may induce feedback oscillation, creating artificial high frequency roughness not present in the true topography of the sample. Although these feedback issues are always present in SPM experiments and are generally solved in an intuitive way, they are specially relevant if the PSD of surface roughness is determined. Indeed, for its faithful determination each image point of the topography raw data has to be acquired faithfully. For rough surfaces--those for which a PSD analysis is particularly interesting--this is a very difficult experimental challenge: feedback parameters have to be adjusted for fast feedback but avoiding oscillations and the imaging speed has to be chosen to allow correct settling of the feedback at each image point (what is "correct" in this context?). In principle, large enough acquisition times (low acquisition speeds) would allow correct measurement of topographic data; however, for large images (more than 10^3 ^× 10^3 ^= a million data points!) this may result in unpractical acquisition times for a single image (up to days!). Moreover, large acquisition times would induce additional problems due to low frequency noise and drift, which would also distort the real topography of the sample.

In this study, we will address in detail how to deal with finite feedback response. We will show that the interaction signal (error signal) can be used not only to quantitatively control but also to significantly improve the quality of the topography raw data used for the PSD analysis. First, we will investigate the effect of feedback response on the determination of the PSD. We will find that, unfortunately, the PSD strongly depends on the setting of the feedback loop (proportional and integral parameters, *P*/*I*) as well as on the imaging speed. Second, we will show that if the error signal used for the feedback is appropriately calibrated, then the correct determination of the PSD can be significantly improved. In particular, the calibrated error signal can then be used in combination with the topography image for a faithful determination of the surface morphology from which the correct PSD measurement is obtained. Moreover, the corresponding PSD curve is much less affected by the fine-tuning of feedback parameters, and allows for much faster image acquisition speeds without loss of information in the PSD curve.

## 2 Experimental and data processing

Experiments were performed using a NanoTec SFM system composed of SFM head, high voltage controller and PLL/dynamic measurement board [30]. In this kind of experiments we use sharpened tips with a force constant of 2 N/m and a resonance frequency around 70 kHz [31]. In order to obtain maximum stability of the mechanical set-up, the microscope was kept working overnight before the relevant measurements were performed. For the experiments discussed in this study we operate the SFM in dynamic mode using the oscillation amplitude as feedback channel (AM-DSFM). Relatively large oscillation amplitudes (50-80 nm peak-peak) and significant reduction of oscillation amplitude are used for feedback (setpoint of 0.5-0.75 *a*_free _with *a*_free _the free oscillation frequency). We note that, contrary to typical AM-DSFM operation in air, a phase locked loop within the dynamic measurement board is enabled to keep the tip sample system always at resonance.

A precise calibration of oscillation amplitude (better than 5%) is essential for the study discussed here. Thermal noise is used to precisely calibrate the oscillation amplitude. We have recently shown how the amplitude signal, and in particular the thermal noise of the amplitude signal, is processed by the electronics of a dynamic unit [32]. Essentially the dynamic unit demodulates the thermal noise signal, therefore the spectrum of the amplitude (and/or phase) can be acquired either with a signal analyzer, or from the fourier transform of the time domain signal of the oscillation amplitude. From the equipartition theorem *kT*/2 = *c < a*^2 ^*>*/2 (*kT *is the thermal energy and *a*^2 ^the square of the rms amplitude signal) the amplitude signal is calibrated if the force constant *c *of the cantilever is known.

The PSD of an image is usually calculated from the 2D Fourier Transform of a topographic image by angle averaging the Fourier transform in all directions [2]. Another possibility is to compute the 1D PSD of each (horizontal) line of an image and then average the power spectrums obtained from all lines of the image. The latter approach is used in this study and will be discussed in detail elsewhere. PSD curves have been computed using a specifically programmed Mathematica^® ^code or directly within the WSxM^® ^software [10,33].

## 3 Effect of feedback response on the determination of surface morphology and PSD of surface roughness

**Figure 1:**
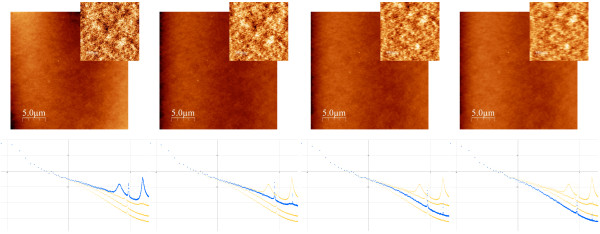
**Topographic images of a glass cover slide acquired with the same imaging speed**. Top row: topography images of the surface of a commercial glass cover slide acquired at a scan frequency of 1 line/s, but at different settings for the feedback loop. Feedback response was decreased from left to right: proportional/integral parameters are 125/25, 80/16, 40/8, and 20/4 (in arbitrary units). Image size in points is 1024 × 1024 and the total acquisition time was about 18 minutes. The insets shows enlarged regions of each topographic image with an amplified grey scale. Lateral size of the larger images is 25 *μ*m, smaller images show a zoom of 5 *μ*m. The total grey scale of all large scale images is 8 nm and 1 nm for all insets. Bottom row: PSD curves calculated from the topographic images; the graphs show the logarithm of the PSD of surface roughness plotted versus the logarithm of the inverse length scale. For each graph the thinner yellow lines show the PSD curves of all the other images, while the thicker blue line shows the PSD curve for the correspondent topographic image shown in the same column. The grid lines for the PSD graphs are Δlog[*κ*] = 1 (horizontal axis) and Δlog[PSD] = 1 (vertical axis).

To illustrate the problem of feedback response on the determination of the PSD curve, Figure 1 shows a series of topographic images of a glass cover slide. Images were acquired with the same imaging speed (1 line/s) but different proportional/integral (*P*/*I*) feedback parameters. The cover slide has been carefully cleaned in order to remove any contaminations from the surface. We note in this context that already a small number of contamination particles ("nanoscale dust") on the glass surface will change the PSD curve obtained from an experimental image, and thus affect the statistical properties of the measured surface morphology. Such a fractal surface was considered particularly appropriate for the present study, since it will have surface roughness at all length scales. We recall that an ideal self-affine surface will show a linear relation of the surface roughness in a log-log plot: log [PSD(*λ*)] versus log(*λ*) = *s *· log(*λ*) + *b*. For the case of the PSD curves shown (averages of the PSD curves for each line), a fractal dimension *D*_f _= 1.5 should result in a slope *s *= 2*D*_f _*- *5 = *-*2 [2]. In addition, a self-affine surface should present a characteristic disordered appearance having large as well as small scale structures. More precisely, it should have larger "large scale structures" and smaller "small scale structures". In our experiments, this "cloudy" appearance is best recognised in the insets of the larger topographic images.

**Figure 2:**
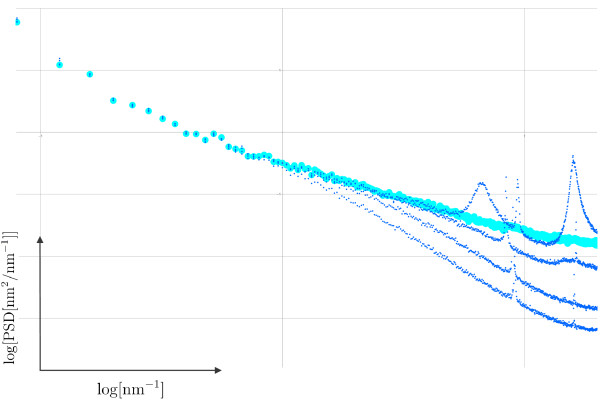
**PSD curves calculated from the topographic images shown in Figure 1**. The graphs show the logarithm of the PSD of surface roughness plotted versus the logarithm of the inverse length scale. The thinner lines correspond to the PSD curves of the individual images shown in Figure 1, the thicker line to a master curve as described in the main text. The grid lines for the PSD graphs are Δlog[*κ*] = 1 (horizontal axis) and Δlog[PSD] = 1 (vertical axis).

Figure 2 shows all PSD curves calculated from the different topographic images shown in Figure 1. In addition, a master curve is shown for comparison, which gives the "true" PSD, to be discussed in detail below. As expected from the arguments discussed in the introduction, images acquired with different setpoints of the feedback loop indeed result in quite different PSD curves. The PSD curve obtained from the image with the highest *P*/*I *values of the feedback loop gives the highest values for the surface roughness. This PSD curve shows three clear peaks, which are only recognised in the PSD curve but not in the corresponding topographic image, where they are essentially imperceptible even in the zooms of the large scale image. As the *P*/*I *values are decreased, the measured surface roughness also decreases. The measured curves do not vary in a simple way since the surface roughness is "lost" differently for large and small length scales. In particular, the three peaks observed in the "fasted" image, strongly decrease when the *P*/*I *values are decreased. Moreover, the "loss" of surface roughness affects the overall shape of the curve, and in particular its slope, from which the fractal dimension is determined. Finally, we note that the "cleanest" curves with a relatively linear shape are obtained for the lowest values of the *P*/*I *parameters. Intuitively we would expect the middle curves to be the better ones because low frequency components are not lost (too much?), and no high frequency components are "produced" due to feedback oscillations. However, how can we assure that this argument is correct? Can we define precise criteria in order to choose the correct PSD-curve? To address this issue, in the next section we will present a simple model in order to relate the measured topography with the true topography and the measured tip-sample interaction. Nevertheless, and in order to stress the importance of this issue, Figure 2 shows a "master curve" representing the true PSD curve of the glass cover slide. This "master curve" will be discussed in detail below. We note that--quite disturbingly--none of the PSD curves obtained from the measured images coincides with the correct "master curve".

## 4 Simple modeling of the imaging acquisition process

The key idea of this section is that topography and error signal should be complementary if appropriate measuring units are utilized. Essentially, this is the key point of the present study. Note that if feedback is slow so that small scale features are filtered in the topography image, these features will appear in the signal that is used to maintain a constant tip-sample inter-action (error signal). On the contrary, if feedback were perfect--which is unphysical--the interaction signal would be constant and all information would be in the topographic image. Finally, if the feedback oscillates, this oscillation should be visible in both, in the topographic and in the error signal image. In order to analyze this point further, we recall that topography images are acquired by maintaining constant the interaction between tip and sample as the sample is scanned; that is, the feedback should fulfill the mathematical condition

(1) $$I\left(x,y,z\left(x,y\right)\right)=I_{\text{set}}$$

where *I*_set _is the setpoint for the feedback, and *z*(*x, y*) is the surface profile followed by the tip. For a given interaction field *I*(*x, y, z*), the SPM system therefore "solves" the implicit equation (1) for the surface profile *z*(*x, y*). In most cases the force field is complicated and highly nonlinear and may even depend on the chemistry of the sample [34]. Then the surface profile depends in a non-trivial way on the set point chosen for image acquisition: *z*(*x, y*) = *z*(*x, y, I*_set_). To keep the present analysis simple, we will assume that effects due to non-linearity and surface chemistry are not relevant for the experiments discussed here, that is, we will assume that for a (reasonable) variety of setpoints the measured profile does not depend on the setpoint chosen for the feedback loop.

For a real, non-ideal feedback loop the surface profile *z*_fb_(*x*(*t*)) followed by the tip of the SPM system will deviate from the true surface by some error profile *δz*_err _(*x*(*t*)):

(2) $$z_{\text{fb}}\left(x\left(t\right)\right)=z_{\text{true}}\left(x\left(t\right)\right)+\delta z_{\text{err}}\left(x\left(t\right)\right)$$

where we have assumed that only the fast scanning direction *x *is relevant for the present discussion and have thus omitted the slow scan direction *y*, because for the *y *direction the feedback loop has sufficient time to settle. In order to keep the notation simple, in what follows we will also omit the time dependence of the signals. Note, however, that this dependence is quite important since a faster scan *x*_fast_(*t*) will imply more error signal and a different surface profile *z*_fb_(*x*_fast_(*t*)).

For a given surface profile *z*_fb_(*x*) the interaction signal which is measured will be

(3) $$\begin{array}{ccc} I_{\text{meas}}\left(x,z_{\text{fb}}\left(x\right)\right) & =I\left(x,z_{\text{true}}\left(x\right)+\delta z_{\text{err}}\left(x\right)\right) & \\ & =I\left(x,z_{\text{true}}\left(x\right)\right)+\frac{\partial I}{\partial z}\text{(}x\text{,}\;z_{\text{true}}\left(x\right))\;\delta z_{\text{err}}+\cdot \cdot \cdot \\ & ≃I_{\text{set}}+\Delta I_{\text{err}}\left(x,z_{\text{true}}\left(x\right)\right) \end{array}$$

where we have kept only linear terms in the expansion of the interaction field *I*(*x, z*). In a real experiment, the measured interaction signal *I*_meas _therefore deviates from the chosen setpoint I_set _by the error signal Δ*I*_err _defined above. This deviation is caused by a finite feedback response which results in a time lag between the "ideal" height of the tip (*z*_true_(*x*)) and the height which is reached by the feedback loop (*z*_fb_(*x*)). Since the tip moves over the surface, by the time the feedback would have settled to the correct height the tip is at a new (lateral) position *x *+ *δx *where the tip-sample height *z*_true_(*x *+ *δx*) in general will be different and the height *z*_fb_(*x *+ *δx*) found by the feedback loop is, again, not correct. Therefore, a SPM system does not measure the real topography *z*_true_(*x*), but some other profile *z*_fb_(*x*). As discussed previously, the amount of error will depend on the scan speed. For linear systems the error is expected to be proportional to the scan speed. An important consequence of relation (3) is that the error profile *δz*_err_(*x*) can be obtained if the "calibration factor" ∂*I*/∂*z *is known:

(4) $$\delta z_{\text{err}}=\Delta I_{\text{err}}\left(x\right)/)\frac{\partial I}{\partial z}\left(x,z_{\text{true}}\left(x\right)\right)$$

With this error profile, the true topography can be obtained directly from the measured topography *z*_fb _and the error signal Δ*I*_err_:

(5) $$\begin{array}{ccc} z_{\text{true}} & =z_{\text{fb}}-\delta z_{\text{err}}= & \\ & =z_{\text{fb}}\left(x,I_{\text{set}}\right)-\Delta I_{\text{err}}\left(x\right)/)\frac{\partial I}{\partial z}\left(x,z_{\text{true}}\left(x\right)\right) \end{array}$$

Unfortunately the slope of the interaction is a quantity which is not easy to determine. Moreover, as discussed above, generally the interaction is nonlinear, therefore its slope will vary with tip-sample distance and thus with the set point chosen for constant interaction images. There are, however, two important SFM modes where the interaction signal can be calibrated appropriately and where the error signal depends linearly on tip-sample distance for a suitable range of tip-sample interaction: the normal force signal in the case of contact mode SFM and the oscillation amplitude in the case of the so called AM-DSFM mode. Moreover, in these SFM modes the interaction signal can be calibrated appropriately so that the error signal can be specified directly in length units (nanometers); that is, the interaction signal is then normalized so that conversion factor ∂*I*/∂*z *is unity, thus Δ*I*_err _= *δz*_err_. For contact mode SFM the interaction signal is then essentially the (static deflection) of the cantilever, while in case of AM-DSFM mode the interaction signal is the oscillation amplitude of the cantilever. In these two SFM modes the true topography is directly obtained by simple substraction of the topography and error signal data:

(6) $$z_{\text{true}}=z_{\text{fb}}-\Delta I_{\text{err}}$$

Therefore, from the topographic and the error image the true topography of a sample can be obtained, which is the raw data for the precise determination of the PSD curve. Evidently, the true topography does not depend on the particular set of parameters used for the feedback loop. When the experimentally measured topography and the error signals are considered uncorrelated entities, both will depend in a very strong way on these parameters. Experimentally--as shown in the following section--only the combination of both, topographic and error signal, is therefore a quantity (i.e., the true topography) that does not depend on a particular set of parameters used for data acquisition (proportional/integral parameters, scan speed, etc.).

## 5 Determination of true surface morphology using topographic and error signal data

**Figure 3:**
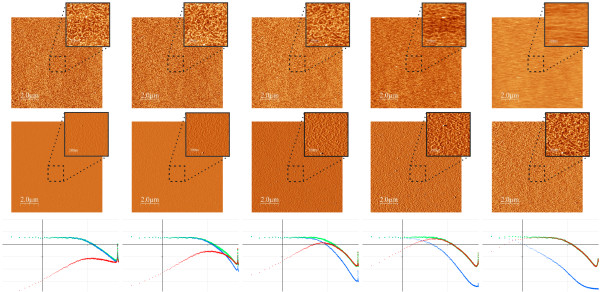
**Images of a Platinum surface taken all at a scan frequency of 1 line/s, but at different settings for the feedback loop**. From left to right, feedback parameters settings are (in arbitrary units): 90/45, 45/22.5, 15/7.5, 5/2.5 and 1/0.5. The images and graphs in the same column correspond to a common data set, since the corresponding images have been acquired simultaneously at a fixed values of the feedback loop. The upper row shows topographic images, the middle row amplitude images and the lower row graphs of the PSD curves of surface roughness. In each graph, the PSD of surface roughness has been calculated for the topography and amplitude image shown in the corresponding column, as well as for the difference *z*_true _= *z*_fb _*- *Δ*I*_err _(corresponding image not shown). Lateral size of the larger images is 10 *μ*m, smaller images show a zoom of 1 *μ*m. The total grey scale of all images is 5 nm (large and small scale images as well as topographic and amplitude images). Lower row: PSD curves calculated from the topography and amplitude images as well as the difference data (image not shown). The grid lines for the PSD graphs are Δlog[*κ*] = 1 (horizontal axis) and Δlog[PSD] = 1 (vertical axis). Green lines correspond to the PSD curves of topography plus error signal, blue curves to the topography and red curves to the error signal.

To illustrate the issues discussed in the preceding section, Figure 3 shows a series of images of a thin Platinum film evaporated onto a Silicon surface. Such a film presents a grain like structure, with a typical lateral grain size of 50 nm. This is precisely the reason why the sample has been selected for this section: as shown in more detail below, the grain like structure results in a well defined surface morphology with a flat distribution of roughness for small inverse wavelengths, and a fast decay for high inverse wavelengths.

This behavior will significantly simplify the precise analysis of how the finite feedback response determines the structure of the measured topography and error signal data. The Platinum grains can be clearly resolved in the enlarged areas of most images. Topography as well as the corresponding amplitude (= error signal) image are presented. As discussed in the experimental section, the amplitude images have been carefully calibrated in order to determine the precise oscillation amplitude. This allows to show the amplitude images in length units (nm). Therefore all data--topography as well as amplitude images--can be shown with the same units and the same scale, in this case 5 nm. Images have been acquired at a scanning rate of 1 line/s, but with different feedback parameters: the images in the first column have been acquired with the "fastest" feedback (high values for the proportional/integral parameters) while those in the last column have been acquired with the "slowest" feedback parameters. Correspondingly, the Platinum grains are visualized in the topography (top row) when the feedback is "fast", and in the amplitude (middle row) when the feedback is "slow". Note that the first topography image is essentially equivalent to the last amplitude image, that is, visually the contrast of the grains is the same. This proves that the calibration of the amplitude image is correct, otherwise the height of the grains would appear different in the topography and the amplitude image (recall that the grey scale of the images is the same for all images).

In addition to the topography and error signal images, for each data set PSD curves have been computed for both signals individually as well as for the difference *z_d_*(*x, y*) = *z*_fb_(*x, y*) *- a*(*x, y*). As expected from the discussion in the previous section, PSD curves obtained from the individual topography and error signal images strongly depend on the feedback parameters. The PSD curves of the difference image give always, within our experimental error, the same "master PSD curve".

Before further discussing the different PSD curves we note that contrary to the case of the glass slide the ("good") PSD curves of the Platinum grains are not linear, instead they saturate for low spatial frequencies (large scales). In order to understand this behavior, we propose a simple model for the morphology of this surface: a disordered arrangement of individual gaussian grains with a fixed height *h*_0 _and a fixed lateral dimension *w*_0_,

$$z_{pt}\left(x,y\right)=h_{0}\;e^{-\left(x^{2}+y^{2}\right)/\left(2w_{0}^{2}\right)}$$

In our case, this assumption is not based on any profound insight on the sample, we have chosen this shape because it is smooth on the top and on the bottom of the grains^*a *^and because its Fourier Transform is directly evaluated,

$$FT\left[z_{Pt}\right]\left(k_{x},k_{y}\right)=\frac{1}{2\pi }\iint dxdy\;e^{\iota \left(k_{x}x+k_{y}y\right)}z_{Pt}\left(x,y\right)=\frac{h_{0}}{\kappa_{0}^{2}}e-\left(k_{x}^{2}+k_{y}^{2}\right)/\left(2\kappa_{0}^{2}\right)$$

where *k_i _*= 1/*λ_i _*is the spatial frequency in each direction (*i *= *x *or *y*), and *κ*_0 _= 1/*w*_0 _is the spatial frequency associated to the width of the grains. For a disordered array of *N *grains we expect an "incoherent" contribution of each grain to the total PSD, and if the grains cover the surface in a dense arrangement, we expect about one grain in a cell of width 2*w*_0_, therefore the total number of grains is $N≃{\left(\text{Scan}\;\text{Size}/2w_{0}\right)}^{2}=\text{Area}/\left(4w_{0}^{2}\right)$. The curve log [PSD(1/*λ*)] versus log(1/*λ*) should therefore show a flat region for small spatial frequencies up to the frequency 1/*w*_0 _corresponding to the width of the grains, and a decrease for higher frequencies. Since $e^{-x^{2}}$ decreases faster than any (inverse) power, this decrease is non-linear, that is, the magnitude of the slope of the log [PSD(1/*λ*)] versus log(1/*λ*) curve increases for high frequencies. Correspondingly, this surface is not self-similar and the notion of fractal dimension is not defined.

**Figure 4:**
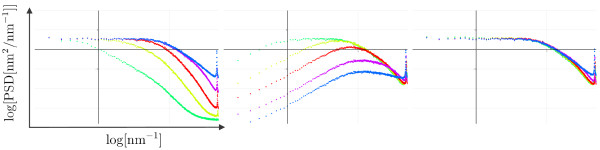
**PSD curves corresponding to the topography and amplitude images shown in Figure 3 as well as of the difference data**. Each graph shows the log (PSD[1/*λ*]) versus log(1/*λ*) curve for the data acquired at different *P*/*I *values. While the different curves are clearly distinguished in the topography **(a) **and amplitude, **(b) **PSDs, the curves corresponding to the difference, **(c) **essentially fall on the same "master curve". The grid lines for the PSD graphs are Δlog[*κ*] = 1 (horizontal axis) and Δ log[PSD] = 1 (vertical axis).

This simple model correctly describes the "good" PSD curves shown in Figures 3 and 4. Moreover, with this simple model for the surface morphology the behavior of the topographic and amplitude images can be further analyzed. In this context we recall that spatial (*k*) and temporal frequencies (*ν*) are related through the scan speed *v*:

(7) $$k=\frac{1}{v}\nu$$

In the graphs shown, the highest and lowest spatial frequencies (0.04 *μ*m^-1 ^and 20 *μ*m^-1^) correspond to a temporal frequency of 1 kHz and 2 Hz.

A characteristic feature of the amplitude PSD curves shown in Figure 3 is that for low spatial frequencies, all curves have a constant slope *s *= 2.0 *± *0.1. Since for these spatial frequencies the PSD curve of the true topography is constant, we conclude that the slope of the PSD curve is determined by the filtering properties of the feedback loop. Indeed, if the *P*/*I *controller is modeled by a simple first order electronic circuit with characteristic time *τ*_0 _we expect transfer functions

(8) $$g_{\text{topo}}\left(\nu \right)=\frac{1}{1+i\tau_{0}\nu }\;\;\text{and}\;g_{\text{amp}}\left(\nu \right)=1-g_{\text{topo}}\left(\nu \right)=\frac{i\tau_{0}\nu }{1+i\tau_{0}\nu }$$

for the topographic and the amplitude signals. Therefore, for low frequencies the amplitude signal will grow linearly with frequency up to the characteristic frequency 1/*τ*_0_. The power of the amplitude signal increases quadratic with frequency and the log [PSD[amplitude(1/*λ*)]] versus log(1/*λ*) curve should give a straight line with slope *s *= 2, as is indeed observed experimentally.

For this sample with constant PSD of surface roughness up to the characteristic spatial frequency *κ*_0 _= 1/*w*_0 _the PSD of the amplitude signal is therefore easily understood taking into account the transfer function of the feedback loop. A similar analysis for the topographic signal is less evident, because in the frequency range where the topographic signal is filtered (for high frequencies) the PSD curve of the true surface roughness does not follow a simple relation (constant or linear). Nevertheless, as the *P*/*I *values are decreased, the topographic signal is clearly filtered more strongly. Moreover, as the *P*/*I *values are decreased for the different set of images, the characteristic frequency 1/*τ*_0 _of the feedback loop also decreases (spatial frequencies 12.2, 12.2, 8.7, 5, and 2.3 nm^-1 ^for *P*/*I *90/45, 45/22.5, 15/7.5, 5/2.5, and 1/0.5, respectively, in Figure 4). Finally we note that even though topographic and amplitude PSD curves are quite different for each set of *P*/*I *values, the PSD curves of the difference image give always, within our experimental error, the same "master PSD curve". This is recognized most easily in Figure 4, where all PSD curves corresponding to the same kind of data (topography, amplitude and difference data) have been collected in the same graph in order to directly visualize how these curves vary as the *P*/*I *values are changed. Very clearly the topographic and amplitude signals vary, but the difference signal is constant. We stress that what seems to be a single curve in Figure 4 is the superposition of the five sets of difference data obtained from topographic and amplitude data shown in Figure 3.

**Figure 5:**
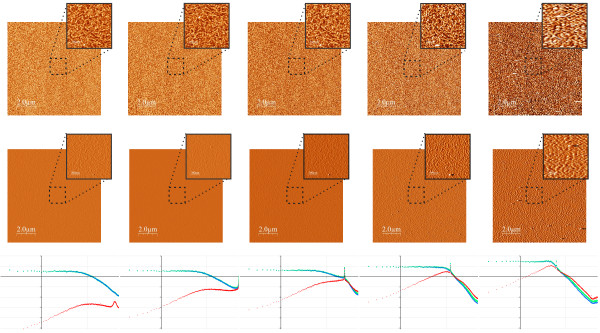
**Images of the same Platinum film as shown in Figure 3**. The images shown were acquired at the same value of the feedback loop, but at different imaging speeds (from left to right: 0.5, 1, 2, 4, and 8 lines/s). As previously the upper row shows topographic images, the middle row amplitude images and the lower row graphs of the corresponding PSD curves of surface roughness. Again, lateral size of the larger images is 10 *μ*m, smaller images show a zoom of 1 *μ*m. The total grey scale of all images is 5 nm. The grid lines for the PSD graphs are Δlog[*κ*] = 1 (horizontal axis) and Δlog[PSD] = 1 (vertical axis).

Figure 5 shows a similar data set as that shown in Figure 3, however in this experiment instead of varying the *P*/*I *values, the scan speed is varied (from left to right: 0.5, 1, 2, 4, and 8 lines/s). We first note that as the scan speed is increased, a characteristic peak moves towards lower frequencies. This peak is first observed in the second graph^*b*^, and also in the data of Figure 3 at essentially the same position. We attribute this peak to oscillation of our system rather than to a true topographic feature. Therefore, according to relation (7) as the scan speed is increased, higher temporal frequencies are measured and the (relative) position of the peak shifts towards lower values.

As compared to the data shown in Figure 3, only the first two data sets give "nice" PSD curves. For high scan speed, the topography and amplitude images do neither result in "clean" PSD curves, nor does the difference data obtained from each set of images result in a PSD curve that is independent of scan speed; that is, the difference PSD curves do not lay on a single "master curve". In the case of the images shown in Figure 5 we find that data which corresponds to frequencies higher that the peak shows a behavior which is not compatible with the simple first order model of the feedback loop discussed above. This model essentially predicts a well defined distribution of topographic and amplitude signal as a function of frequency according to relation (8). In particular, the PSD curves of the topographic data acquired at the faster frequencies do not decrease more strongly than the amplitude data, which should be the case if the assumption leading to relation (8) were strictly valid. Note that in this region, the amplitude signal is no longer high pass filtered (the amplitude signal is "over its maximum", and this maximum defines the characteristic frequency 1/*τ*_0 _of the feedback loop), which implies that the topographic data should be high pass filtered. However, we observe no high-pass filtering of topography data in these data sets (compare this region of the PSD curves with the corresponding behavior in Figure 3). We attribute this non-standard behavior to the fact that at these high temporal frequencies the SPM setup cannot be considered a simple (electronic) first order system defined only by the *P*/*I *values of the feedback loop. Instead, also mechanical resonances of the SPM setup and possibly even non-linearities of the tip-sample interaction have to be taken into account, rendering the tip sample system a much more complicated system in terms of transfer characteristic. In particular, we believe that for a faithful description of the tip-sample system orders higher than one, and possibly also nonlinearities, have to be taken into account.

**Figure 6:**
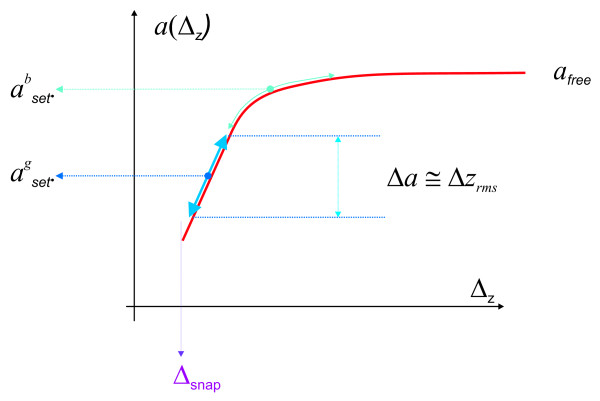
**Schematic representation of the oscillation amplitude versus the tip-sample distance**. For large tip sample distances, the free oscillation amplitude is measured. As the oscillating tip interacts with the surface the oscillation decreases linearly with tip sample distance. For small oscillation amplitude, the energy pumped into the cantilever by the external driving circuit is not sufficient to compensate for the losses induced by the tip-sample interaction, and the oscillation stops. If the feedback loop does not respond instantaneously to height variations as the tip is scanned over the surface, height variations will result in variations of the oscillation amplitude. For the kind of applications proposed in this study, the tip-sample system has to stay in the linear regime of the amplitude versus distance curve.

Finally, some additional practical issues not discussed so far should be emphasized. First, we note that for each SFM system the correct polarity of the error signal will have to be determined (what is seen low/high by the error signal?). This polarity determines the sign of relation (6), that is, whether the error signal has to be added or subtracted (as assumed in this study) in order to obtain the "true" topography. A second issue not discussed yet is the correct setpoint for image acquisition. In order for the error signal to faithfully reproduce the surface morphology, it has to be a linear function of tip-sample distance. Therefore the setpoint has to be chosen so that there is "enough signal" when passing over high and low surface features. This is illustrated in Figure 6 for the case of the oscillation amplitude as error signal. If possible, the setpoint *i*_0 _of the interaction should be chosen such that the values between *i*_0 _*- δz*_rms _and *i*_0 _+ *δz*_rms _depend linearly on the tip-sample distance (recall that the error signal is calibrated in length units), where *δz*_rms _is the rms roughness of surface morphology. If the setpoint *i*_0 _is chosen too close either to the free oscillation amplitude ($a_{\text{set}}^{b}$ = bad setpoint in Figure 6) or to the minimum amplitude needed to sustain a stable oscillation, then a small surface roughness will move the tip-sample system from the linear part of the amplitude versus distance curve. If this is the case, the error signal will not any more contain the correct information about the surface morphology, and the true surface morphology cannot be reconstructed as discussed in this study.

## 6 Conclusions

We have shown that the correct determination of surface morphology using SPM techniques is extremely demanding and may be easily affected by experimental parameters. The description of surface morphology using PSD curves is a very powerful tool, but requires very good experimental raw data. In order to exploit the full potential of PSD analysis every experimental data point has to be a faithful representation of the true surface. For feedback based and sequential imaging techniques such as SPM, this is a very difficult task. The data shown here proves that in most cases if only topography data is acquired, the measured morphology is significantly affected by the feedback response of the SPM system. Then, the PSD curves calculated from this experimental data do not correspond to that of the true topography. Instead, either features are "lost" due to low pass filtering or features are "created" due to oscillation of the feedback loop. In most cases the PSD curves obtained from topographic images depend strongly on the parameters used for data acquisition (scan speed, *P*/*I *values of the feedback loop), and it is not clear which, if any, is the "good" curve. When the error signal of the feedback loop is acquired and analyzed, the characteristic response time of the feedback loop can be determined. This response time determines, together with the scan speed, the maximum spatial frequency up to which the topographic data is measured faithfully. In addition, possible oscillation of the feedback loop can also be recognized in the error signal. The error signal can thus be used to control the quality of the topographic image.

If the error signal is correctly calibrated (in length units: [nm]), then the topographic and the error signal can be summed (with the correct sign!) in order to give a "true" image that is a faithful representation of the surface morphology. In particular, this combined image does not depend on the particular set of parameters used for image acquisition. Interestingly, we observe that the best data is not acquired with high feedback parameters, since these result in oscillation of the feedback loop; imperceptible in the topographic and error signal data, but clearly observed in the corresponding PSD curves. Therefore for faithful imaging of the surface, the feedback parameters should not be pushed too high, instead, as discussed in this study, the calibrated error signal should be used to "recover" the small scale surface morphology, which is (low-pass) filtered by the feedback loop in the topographic image.

Since the nanoscale surface morphology determines many surface related processes such as friction, adhesion, wetting as well as many others, its correct determination is a fundamental issue in nanoscience. From a theoretical point of view, the PSD of surface topography is a basic tool to describe the statistical properties of surfaces and is used as a key parameter for the description of surface morphology in modern theories of friction and adhesion. Accordingly, its precise experimental measurement as proposed in this study is a fundamental issue for nanoscience, and we strongly believe that the approach presented in this study substantially improves the performance of any SFM when using contact mode SFM as well as AM-DSFM, which are the two modes used for most imaging applications.

In this study, we have chosen rather large oscillation amplitudes and high reduction of amplitude oscillation (= strong tip-sample interaction). This is no problem for hard surfaces as those discussed in this study, but may be an issue for softer materials. In this latter case, oscillation amplitude as well as reduction of amplitude oscillation will have to be chosen with more care and an optimal compromise between good topography and amplitude data, acquisition speed and sample damage will have to be found. Nevertheless, we believe that the method discussed here will be valuable, since the sum of topography and error data will always reflect better the true topography.

## Competing interests

The authors declare that they have no competing interests.

## Authors' contributions

JFGM performed the SFM measurements. JFGM and INC performed the data analysis and interpretation. JA and JC supervised and coordinated the experiments. JFGM, INC, JA and JC designed and conceived the experiments. Finally, JC wrote the paper. All authors read and approved the final manuscript.

## Endnotes

^a^Note that even for a sharp surface feature the SPM images would be smoothened due to tip convolution, that is, the sharp surface feature would "see" the curvature of the tip.

^b^Probably the wider peak in the first graph has the same origin but is observed wider and at lower frequencies due to aliasing.
